# Triboelectric Enhancement of Polyvinylidene Fluoride Membrane Using Magnetic Nanoparticle for Water-Based Energy Harvesting

**DOI:** 10.3390/polym14081547

**Published:** 2022-04-11

**Authors:** Duy-Linh Vu, Kyoung-Kwan Ahn

**Affiliations:** Fluid Power and Machine Intelligence (FPMI) Laboratory, School of Mechanical Engineering, University of Ulsan, 93, Daehak-ro, Nam-gu, Ulsan 44610, Korea; vuduylinhbk@gmail.com

**Keywords:** magnetic nanoparticle, surface polarization, β phase, dielectric constant, triboelectric nanogenerator

## Abstract

Produced by magnetic material dispersed in a viscous environment for the purpose of collecting and converting energy, magnetic rheological compounds greatly strengthen the development of skin-attachable and wearable electrical equipment. Given that magnetic nanomaterial anisotropy has a substantial influence on the interface polarizing of polyvinylidene fluoride (PVDF), it is critical to explore the function of magnetic polymer compounds in the triboelectric layer of triboelectric nanogenerator (TENG) output power. In this study, ferromagnetic cobalt ferrite, CoFe_2_O_4_ (CFO), nanoparticles, and PVDF were employed to create a triboelectric composite membrane to improve TENG energy output. The content of β phase in PVDF increased significantly from 51.2% of pure PVDF membrane to 77.7% of 5 wt% CFO nanoparticles in the PVDF matrix, which further increase the dielectric constant and negative charge of the membrane. As a consequence, the energy output of CFO/PVDF-5 TENG increased significantly with a voltage of 17.2 V, a current of 2.27 μA, and a power density of 90.3 mW/m^2^, which is 2.4 times the performance of pure PVDF TENG. Finally, the proposal for TENG hopes that its extraordinary stability and durability will provide additional views on hydrodynamic power generation in the future.

## 1. Introduction

In recent decades, water-based triboelectric nanogenerators (TENG) have received a great deal of interest as a potential solution for numerous hydraulic energy harvesting applications, for instance, waves of river and sea [1,2], raindrop [3,4], liquid flow [5,6], etc., due to its high-strength, lightweight, cost-effective, abrasion-resistant, and eco-friendly characteristics [7,8,9]. The TENG device works on a synergy between triboelectrification to generate a surface charge and electrostatic induction to guide the electrode through an external circuit [10,11,12]. The triboelectric layer therefore is critical to the energy performance of the water-based TENG depending on the operating mechanism and structure design. Previous studies have shown several methods aimed at improving efficiency performance by adding auxiliary materials, surface treatments, or structural designs based on various triboelectric properties, such as surface potential, permittivity, and structural properties, which have been investigated [13,14,15]. According to the different methods for triboelectric effect enhancement, ferroelectric is one of the key materials that is lightweight, flexible, high-strength, and has attracted considerable attention. Furthermore, ferroelectric materials specialize in converting mechanical and thermal stimuli into electrical responses due to their special piezoelectric and thermoelectric properties [16,17,18,19].

Unlike inorganic ferroelectric materials, which have hardness, brittleness, and involve tedious manufacturing processes, ferroelectric polymers have flexibility, treatability, and impressive physics, making them promising candidates for wearable and implantable electronic devices for triboelectric devices [20,21]. Polyvinylidene fluoride (PVDF) is suggested as a useful material that can fabricate triboelectric layer for water-based TENG owing to its large polarizability in the crystal structure and strong surface potential [22,23,24]. PVDF is composed of a CF_2_CH_2_ monomer chain, which is arranged into various stages to form the α, β, γ, and ε phases. In general, the α phase outperforms the PVDF phase element due to its remarkable thermodynamic stability. On the other hand, the α phase does not exhibit ferroelectricity due to its nonpolar molecular chain conformation, while the polar crystal phase (β phase) has a zigzag structure with fluorine, and hydrogen atoms are oriented in opposite directions. Furthermore, compared to other polymers, PVDF with a high-phase composition has a greater dielectric constant and a bigger negative charge [16,20,25,26,27]. Therefore, increasing the β phase of the PVDF membrane is a critical point for optimizing the output energy of water-based TENG devices.

The use of all monomer units, such as trifluoroethylene (TRFE), hexafluoroethylene (HFE), hexafluoropropylene (CTFE), and cheroloopropylene (CTFE), through the polymerization process is obtained with the high concentration of β phase. Contrastingly, their methods requires complicated synthetic procedures and high-value ingredients [16,28,29]. Further, due to applying the high-strain force in the polymeric chain, the mechanical modification can increase the β phase. Some advantages of this method are simple procedures, low cost, and mass production, but PVDF membranes continue to suffer a number of issues. After reducing the mechanical strain, the polymer molecular structure might revert to its original state, resulting in a drop in β phase content. In addition, large strains, typically 400–500%, are required to induce atomic phase transitions, which are not suitable for creating composite materials that can successfully combine the benefits of PVDF with other inclusions, especially at high filler content. It leads to uncontrolled reorganization and agglomeration of the filling material [30,31,32]. Another way to enhance the content of the β phase is to add fillers, such as carbon nanotubes (CNTs) [33,34], BaTiO_3_ nanoparticles [35,36], and clays [37], into PVDF to form nanocomposites. However, performance optimization of the TENG devices due to the synergistic effect between ferroelectric polymers and piezoelectric ceramics still needs to be improved.

In this work, we propose a realistic approach to synthesizing a high level of β phase content for a triboelectric layer of the TENG devices by constructing ferromagnetic cobalt ferrite, CoFe_2_O_4_ (CFO), as fillers in the PVDF matrix. Due to the moderately high magnetization, high coercive force, and relatively large magnetic anisotropy, the addition of CFO nanoparticles could significantly increase the content of the β phase in the PVDF membrane, which notably impacts the performance output of the proposed TENG. As calculated from FTIR spectra, the content of β phase increases from 51.2% of the pure PVDF membrane to 77.7% of the PVDF membrane with 5 wt% CFO addition. Moreover, the dielectric constant of the optimized membrane was also increased by 80%. The results demonstrated that the power density of CFO/PVDF-5 TENG reaches a maximum of 90.3 mW/m^2^. 

## 2. Materials and Methods

### 2.1. Fabrication of CFO/PVDF Membrane

**Figure 1:**
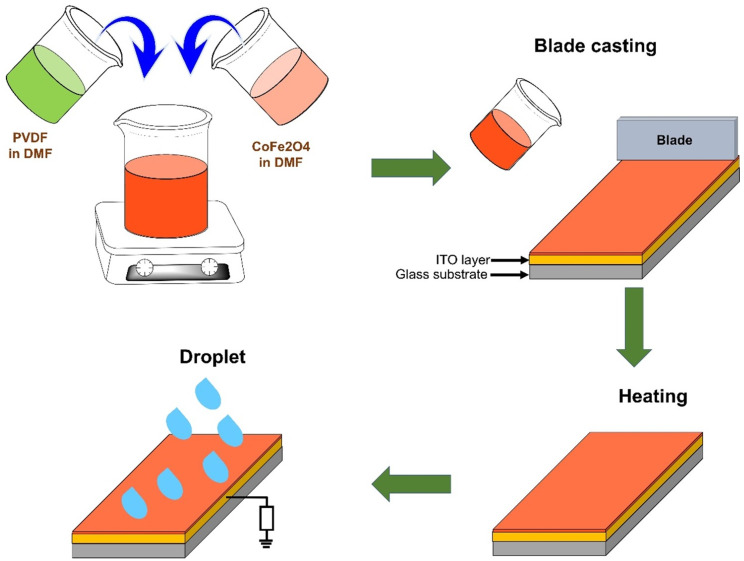
Schematic diagram of CFO/PVDF membrane fabrication.

Figure 1 shows the fabrication of the CFO/PVDF membrane by using a solvent-casting process. First, PVDF powder (*M*_W_~534,000, Sigma-Aldrich, St. Luois, MO, USA) and CoFe_2_O_4_ nanoparticles (CFO, 99%, Sigma-Aldrich, St.Luois, MO, USA) were separately dissolved in *N*,*N*-Dimethylformamide (DMF, 99.5%, Samchun Chemical, Seoul, Korea) to obtain the 10 wt% PVDF and 1 wt% CFO solution. Then, the CFO solution was added to the PVDF solution in various amounts to obtain the desired CFO/PVDF ratio (2.5, 5, 7.5, and 10 wt%). The photography of the PVDF and CFO/PVDF membrane have shown in Figure S1 (Supplementary Materials). After that, the fabricated solution was successfully bladed onto the indium tin oxide (ITO) film (8–12 Ω/sq, Sigma-Aldrich, St. Luois, MO, USA) and dried at 80 °C to ensure DMF evaporation. Each membrane was designated as follows: CFO/PVDF-A, where A is the content of CFO in the PVDF matrix.

### 2.2. Fabrication of CFO/PVDF TENG

The CFO/PVDF TENG device operates through single-electrode mode by using CFO/PVDF membrane as the solid phase, the ITO layer as the electrode, and deionized water as the liquid phase. After coating, the fabricated membranes were cut into a dimension of 2 × 2 cm^2^. The output signals of current and voltage were measured through a copper wire connected to the ITO layer.

### 2.3. Characterizations

Surface morphology was investigated using atomic force microscopes (AFM, MFP-3D Stand Alone AFM, Oxford Instruments, Abingdon, UK) and field emission scanning electron microscopy (FE-SEM, JSM-6500F, JEOL, Tokyo, Japan). In detail, the AFM images were obtained with intermittent-contact mode using Si probes with a nominal normal spring constant of 2 Nm^−1^. Before measuring the FE-SEM image, the membranes were placed on the copper grid, which was coated with carbon film and then covered with a layer of platinum. In addition, the chemical structure of the membrane surface was studied using Fourier transform infrared spectroscopy (FTIR, Nicolet iS5, Thermo Fisher Scientific, Waltham, MA, USA). An impedance analyzer 352250LCR meter (Hioki Electric, Nagano, Japan) was used to investigate the dielectric constant of membranes. A DMM7510 Keithley digital multimeter (Cleveland, OH, USA) was used to measure the output voltage and current of the TENG devices.

## 3. Results and Discussion

### 3.1. Characterization of CFO/PVDF Membrane

**Figure 2:**
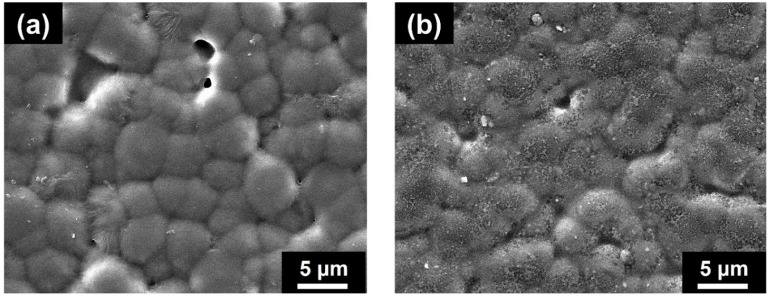
FE-SEM image of (**a**) PVDF and (**b**) CFO/PVDF-5 membrane.

**Figure 3:**
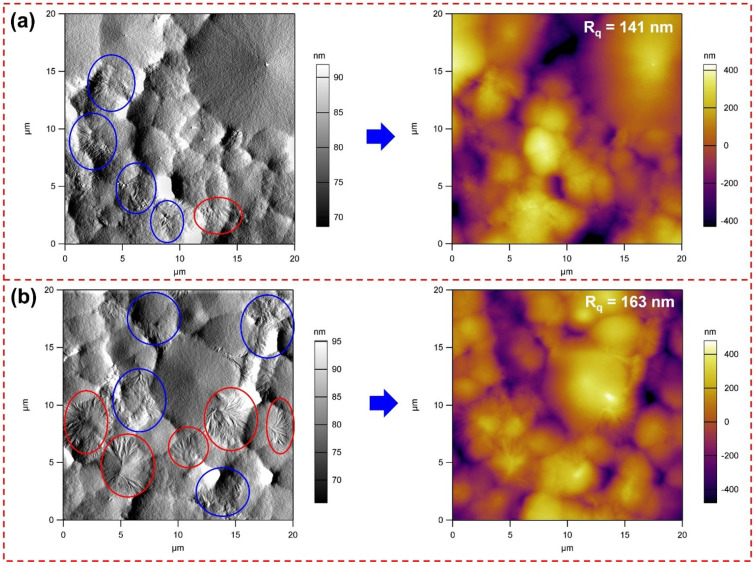
AFM image of (**a**) PVDF and (**b**) CFO/PVDF-5 membrane.

As demonstrated in Figure 2 and Figure S2 (Supplementary Materials), the surface morphology of PVDF and CFO/PVDF-5 membrane was examined with FE-SEM images. Compared to PVDF membranes, CFO/PVDF-5 has a high surface roughness and contains more granular particles owing to the distribution of CFO in the PVDF matrix. In addition, this is due to the phase separation of the CFO and PVDF, which has a substantial impact on the β phase conformation and the dielectric constant of the membrane. Moreover, AFM images (Figure 3) from a near-field microscope were used to clearly distinguish between non-polarity (α) and polarity (β) on the membrane surface [27]. The membrane surface exhibits multiple spherical morphologies corresponding to β (marked by red circles) and β/α hybrid phase regions (marked by blue circles), respectively. In addition, the smooth areas coincide with the α phase of the membrane, which demonstrated that the root mean square roughness (R_q_) of the CFO/PVDF-5 membrane (163 nm) is higher than the PVDF membrane (141 nm) (Figure S3, Supplementary Materials). Therefore, after adding the CFO nanoparticles, the content of the β phase significantly increases. Due to the negatively charged of the CFO nanoparticles, these nanoparticles establish a bond with the positive CH_2_ of PVDF via ionic dipole interactions, with the preferential development of the polar β phase as the result.

**Figure 4:**
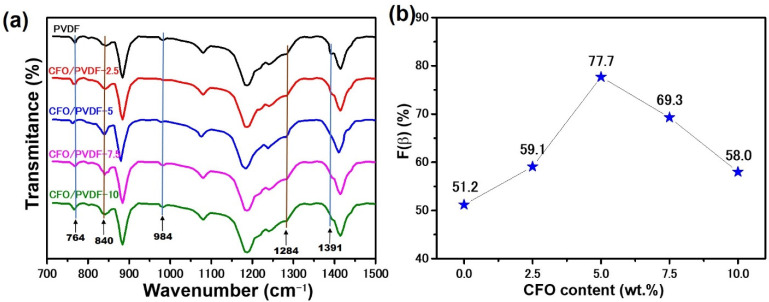
(**a**) FTIR spectra of pure PVDF and CFO/PVDF membranes and (**b**) the corresponding content of β phase.

For more clarity, FTIR was used to quantify the effect of CFO on the phase structure of the PVDF membrane. The α phase is indicated by the peaks at 764, 984, and 1391 cm^−1^, while the β phase is indicated by the peaks at 840 and 1284 cm^−1^, as pictured in Figure 4a, [38]. Suppose that the relative proportions of the β phase, F(β), could be estimated using the measured infrared absorption according to the Lambert-Beer law [39].

(1) $$F\left(\beta \right)=\frac{A_{\beta }}{1.26A_{\alpha }+A_{\beta }}$$

where A_α_ and A_β_ are denoted to the transmittance of α phase at 764 cm^−1^ and β phase at 840 cm^−1^, respectively. The content of the β phase in different PVDF and CFO/PVDF membranes is shown in Figure 4b. Consequently, the addition of CFO nanoparticles dramatically enhanced the content of the β phase. For example, the content of the β phase in the CFO/PVDF-5 membrane reached a maximum value of 77.7%, while the β phase content of the PVDF membrane was 51.2%, indicating the advantage of CFO nanoparticles over the β phase formation of PVDF structure. However, at CFO concentrations above 5 wt%, the content of the β phase begins to decrease. This can be explained by the agglomeration of a high concentration of CFO nanoparticles on the film surface [22].

**Figure 5:**
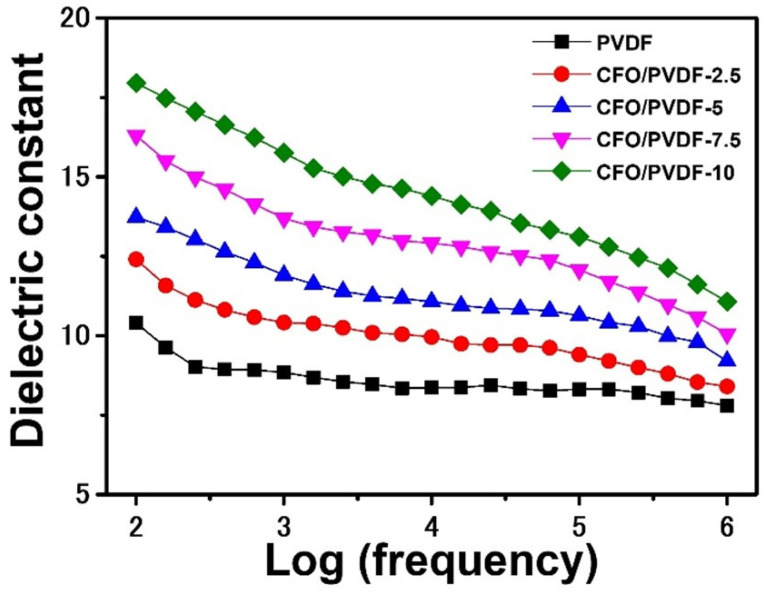
Dielectric constants of pure PVDF and CFO/PVDF membranes at a different frequency.

In addition, CFO nanoparticles are a renowned magnetic material with an excellent dielectric constant that impacts the TENG electrical output [40,41]. As a result, Figure 5 investigates the dielectric constant of the pure PVDF and CFO/PVDF membranes relying on frequency dependence. The pure PVDF membrane has a dielectric constant of 10.4 at 10^2^ Hz and subsequently declines as the frequency increases, demonstrating the same pattern as prior research [20,40]. Furthermore, the dielectric constant of the CFO/PVDF membranes follows the same trend as the pure PVDF membrane in the frequency range of 10^2^–10^6^ Hz and increases as the concentration of CFO increases. Therefore, the dielectric constant of the CFO/PVDF-10 membrane reaches the highest value of 17.9 at 10^2^ Hz, which is 1.72 times larger than the PVDF membrane. 

### 3.2. Characterization of CFO/PVDF TENG Electrical Output

#### 3.2.1. Working Mechanism

**Figure 6:**
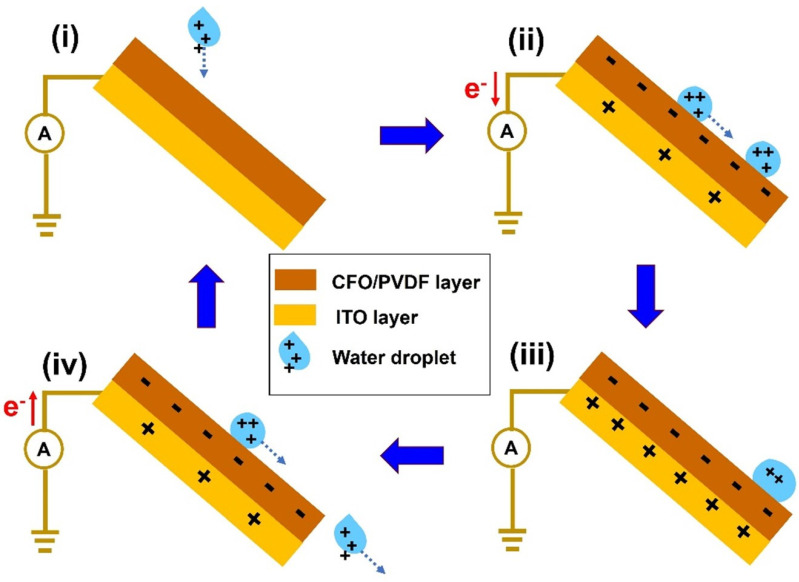
Working mechanism schematic of the water-based TENG.

The working principle of water-based TENG in this study is illustrated in Figure 6 based on triboelectric and electrification effects. In the initial state, the water droplet was pre-charged and contained by air and silicone tubing to obtain the positive charges before approaching the CFO/PVDF membrane surface (Figure 6i) [42,43]. When the positively charged droplet made contact with the CFO/PVDF layer (Figure 6ii), the electrical balance was disrupted, resulting in an electrical potential imbalance between the CFO/PVDF membrane and the ITO electrode. Electrical currents are generated due to the attractiveness of the ITO electrode towards electrons at the ground. Because of the self-balancing property of the potential difference, it tends to return to equilibrium (Figure 6iii). The electron flow then changes direction due to the generation of potential difference as the droplet exits the CFO/PVDF layer (Figure 6iv). In this situation, an alternating current output could be generated until the next water droplet is generated to provide a continuous current.

#### 3.2.2. Electrical Output Performance

Based on the fundamental working mechanism of the TENG, the transferred charge density ($\sigma^{\prime }$) in this device could be presented as follows [23]:

(2) $$\begin{array}{c} \sigma^{\prime }=\frac{\sigma_{0}d_{gap}}{d_{gap}+d_{m}/\varepsilon_{m}} \end{array}$$

where $d_{m}$ and $\varepsilon_{m}$ refer to the thickness and permittivity of the CFO/PVDF membrane, $d_{gap}$ is the gap distance, and $\sigma_{0}$ is the charge density in the equilibrium state. The enhanced polar β phase facilitates charge-carrier migration and increases charge density in equilibrium [44]. Therefore, as a result of the high β phase of the CFO/PVDF membrane, charge accumulation at the solid interface may increase, the static electric field acting on the triboelectric surface may increase, and TENG performance may be further improved. In addition, this calculation shows that when the dielectric constant grows, the transfer charge density of the membrane improves as well, gaining the benefits of TENG electrical output.

**Figure 7:**
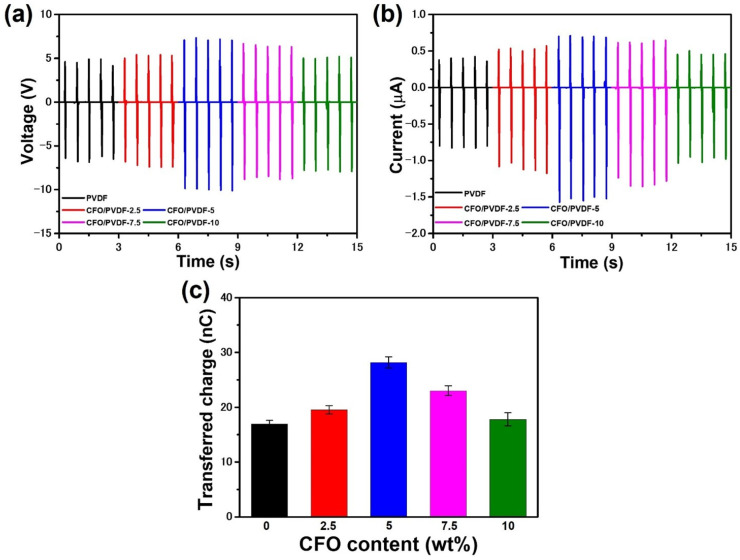
(**a**) Voltage and (**b**) current of PVDF and CFO/PVDF-based TENG; (**c**) comparison of transferred charge of their TENG.

Therefore, characterization of the power output of the pure PVDF TENG and CFO/PVDF TENG is shown in Figure 7 to illustrate how CFO content affects the TENG electrical output. The operating conditions for the TENG are as follows: contact area (A) of 2 × 2 cm^2^, an inclination angle of 45°, DI water droplet size of 50 μL, and dropping height of 10 cm. In general, the electrical output of TENGs exhibits is highly stable, which is advantageous for the operation of electrical systems. Specifically, the peak voltage and current of PVDF-TENG were found about 11.4 V and 1.26 μA, respectively, which seem to be smaller than that of other CFO/PVDF TENG. After adding the CFO nanoparticles, the peak voltage and current of CFO/PVDF TENG were approximately 12.6, 17.2, 14.9, and 12.8 V and 1.67, 2.27, 1.95, and 1.49 μA, respectively, corresponding to CFO content of 2.5, 5, 7.5, and 10 wt%. Similarly, the transferred charge (Figure 7c) of the CFO/PVDF TENG exhibits the same trend as the output current under varied CFO content in PVDF. Notably, CFO/PVDF-5 TENG achieves the maximum electrical output, which enhances the current of 80%, voltage of 51%, and transferred charge of 66% compared to the values obtained for the PVDF TENG. This significant improvement is attributed to greater charge transfer as a result of better β phase composition and dielectric constant, which has been discussed in the above section. However, when the content of CFO nanoparticles exceeds 5% by weight, the output signal of CFO/PVDF TENG starts to deteriorate. This is due to a decrease in β phase content in the CFO/PVDF membrane. Furthermore, high CFO concentration might create agglomeration on the membrane surface, which reduces charge transfer to the surface.

With the goal of demonstrating the capabilities as a power source for electronic devices, the power density of the PVDF and CFO/PVDF-based TENG was examined under various load resistance (*R*) ranges ranging from 1 MΩ to 500 MΩ. The following formula was used to compute the power density (*P*) [45,46]:

(3) $$P=\frac{I^{2}R}{A}$$

**Figure 8:**
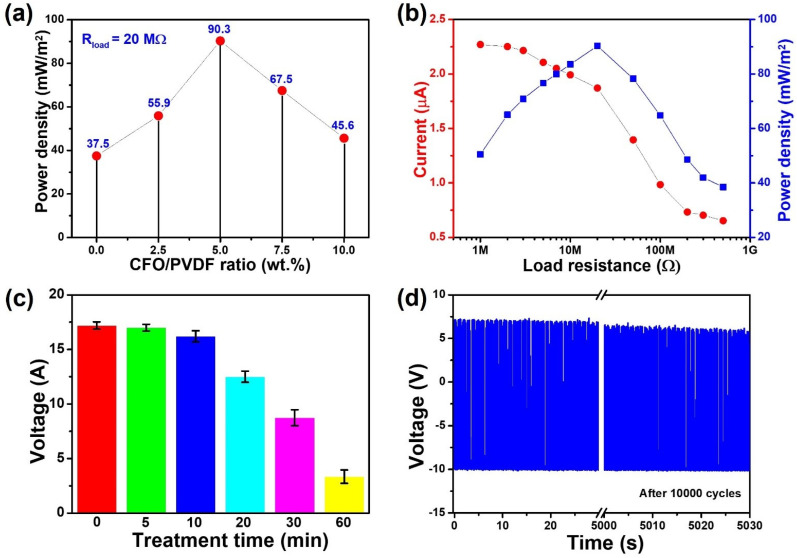
(**a**) Power density of PVDF and CFO/PVDF-based TENG at load resistance of 20 MΩ, (**b**) current and power density of the CFO/PVDF-5 TENG with different load resistance, (**c**) durability of CFO/PVDF-5 TENG following sonication treatment at various periods, and (**d**) stability of CFO/PVDF-5 TENG operated for 10,000 cycles.

As a result, in Figure 8a, the highest power density of CFO/PVDF TENG is 90.3 mW/m^2^ and corresponds to a load resistance of 20 MΩ, which is 2.4 times more than pure PVDF TENG. Moreover, the output current of CFO/PVDF-5 TENG decreases as the loading resistances rise (Figure 8b). Meanwhile, power density increases until a load resistance value of 20 MΩ is reached and then decreases as resistance increases. These findings suggest that the CFO/PVDF-5 TENG system might be used as water-based energy harvesting equipment, where the triboelectric layer made of PVDF and CFO nanoparticles provides better performance.

Furthermore, the high durability and stability of CFO/PVDF-5 TENG are demonstrated in Figure 8c,d. Before being used for the triboelectric layer, the CFO/PVDF-5 membrane was processed to bath sonication at various intervals. The peak voltage of this TENG remained steady after the CFO/PVDF-5 membrane was treated for 20 min, indicating that CFO/PVDF-5 TENG has exceptional mechanical endurance in harsh conditions. Furthermore, the voltage signal remains 90% after working for more than 10,000 cycles, which demonstrates the high stability of the TENG. According to the findings, the CFO/PVDF-5 TENG has a high potential for harvesting water energy in a variety of situations such as rainfall, ocean, wastewater, and so on.

## 4. Conclusions

In conclusion, the electrical output of the water-based TENG was boosted by modifying the crystal morphology and dielectric constant of the PVDF membrane using CFO nanoparticles. Based on the large magnetic anisotropy, the dielectric constant of CFO/PVDF membranes progressively improved when the content of CFO was increased from 2.5 to 10 wt%, and efficiency finally approached over 80% when compared to pure PVDF membrane. More crucially, the content of the β phase improves and reaches the maximum of 77.7% in the CFO/PVDF ratio of 5 wt%. It was attributed to an increase in charge distribution, resulting in greater electrostatic charge movement on the triboelectric interface. As a result, the highest power density of the TENG rises from 37.5 to 90.3 mW/m^2^, which was increased by approximately 2.4 times. Furthermore, the proposed TENG generated by the CFO/PVDF-5 membrane has tremendous potential for mechanical power generation and a self-powered sensor in a hostile environment because of its high durability and stability.

## Data Availability

The authors confirm that the data supporting the findings of this study are available within the article.

## Supplementary Materials

The following supporting information can be downloaded at: https://www.mdpi.com/article/10.3390/polym14081547/s1, Figure S1: Photography of the PVDF and CFO/PVDF-5 membrane; Figure S2: FE-SEM image of (a) PVDF and (b) CFO/PVDF-5 membrane; Figure S3: The measurement parameter of (a) PVDF and (b) CFO/PVDF-5 membrane in AFM image.
