# Supplementary material for: Triboelectric Enhancement of Polyvinylidene Fluoride Membrane Using Magnetic Nanoparticle for Water-Based Energy Harvesting
Source: Polymers (Basel). 2022 Apr 11;14(8):1547. doi: 10.3390/polym14081547 (PMC9026377; doi:10.3390/polym14081547)
Supplement: Supplementary file 1 [file polymers-14-01547-s001.zip › polymers-1655952-supplementary.pdf]

polymers

Supplementary Material

Triboelectric Enhancement of Polyvinylidene Fluoride Mem-brane Using Magnetic Nanoparticle for Water-Based Energy Harvesting

Duy Linh Vu and Kyoung Kwan Ahn *

Fluid Power & Machine Intelligence (FPMI) Laboratory, School of Mechanical Engineering, University of Ulsan, 93, Daehak-ro, Nam-gu, Ulsan 44610, South Korea; vuduylinhbk@gmail.com (D.L.V); kkahn@ulsan.ac.kr (K.K.A)
* Correspondence: kkahn@ulsan.ac.kr

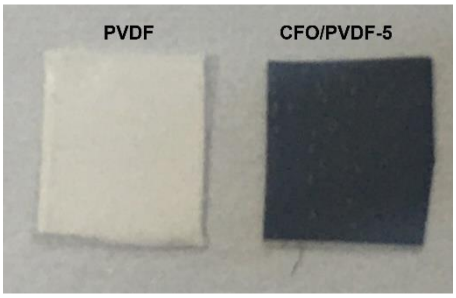

Figure S1. Photography of the PVDF and CFO/PVDF-5 membrane

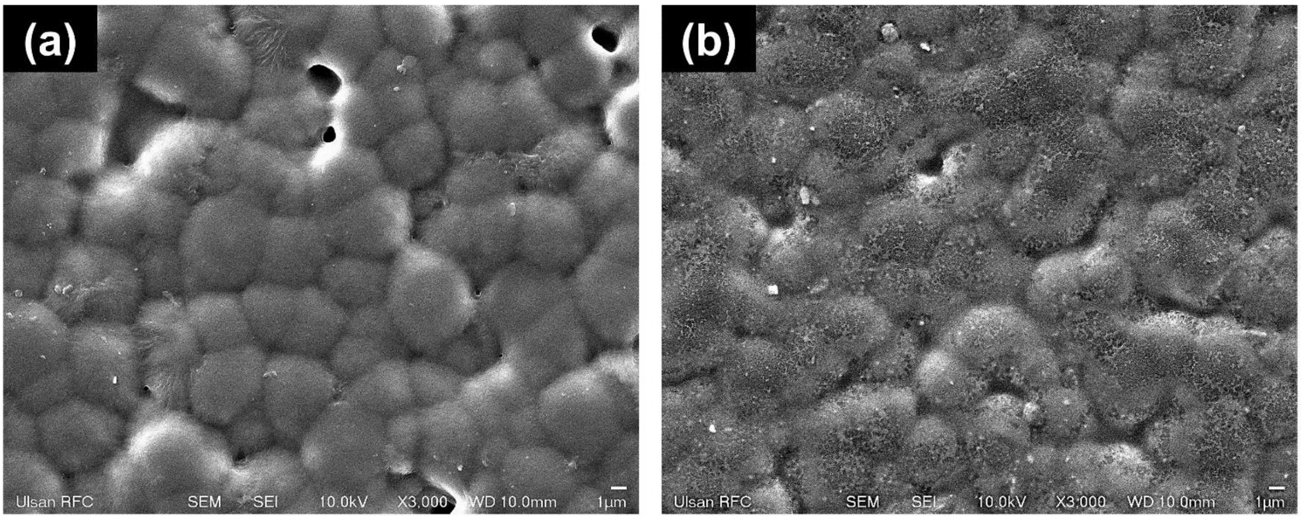

Figure S2. FE-SEM image of (a) PVDF and (b) CFO/PVDF-5 membrane

(a)

Sample1_PVDF membrane
RoughnessData:

Parameter	Full Image Values:	Mask Image Values:
Number Of Points:	262144	
Average:	-0.000 m	
Standard Deviation (Rq):	162.962 nm	
Max:	465.558 nm	
Min:	-573.578 nm	
RMS:	162.961 nm	
Average Deviation (Ra):	133.855 nm	
Skew:	-0.14	
Kurtosis:	-0.464	
Percent XY:	100 %	
Surface Area:	410.0	
Area Percent:	2.492 %	
Volume	-0.0	

(b)

Sample2_CFO/PVDF-5 membrane
RoughnessData:

Parameter	Full Image Values:	Mask Image Values:
Number Of Points:	262144	
Average:	-0.000 m	
Standard Deviation (Rq):	161.108 nm	
Max:	385.066 nm	
Min:	-538.229 nm	
RMS:	161.108 nm	
Average Deviation (Ra):	127.626 nm	
Skew:	-0.345	
Kurtosis:	0.0483	
Percent XY:	100 %	
Surface Area:	406.9	
Area Percent:	1.732 %	
Volume	-0.0	

Figure S3. The measurement parameter of (a) PVDF and (b) CFO/PVDF-5 membrane in AFM image
